# Matrix modification for enhancing the transport properties of the human cartilage endplate to improve disc nutrition

**DOI:** 10.1371/journal.pone.0215218

**Published:** 2019-04-10

**Authors:** Aaron Dolor, Sara L. Sampson, Ann A. Lazar, Jeffrey C. Lotz, Francis C. Szoka, Aaron J. Fields

**Affiliations:** 1 Department of Bioengineering and Therapeutic Sciences, University of California, San Francisco, San Francisco, CA, United States of America; 2 Department of Orthopaedic Surgery, University of California San Francisco, San Francisco, CA, United States of America; 3 Department of Preventive and Restorative Dental Sciences, University of California, San Francisco, CA, United States of America; 4 Department of Epidemiology and Biostatistics, University of California, San Francisco, CA, United States of America; University of Pennsylvania, UNITED STATES

## Abstract

Poor solute transport through the cartilage endplate (CEP) impairs disc nutrition and could be a key factor that limits the success of intradiscal biologic therapies. Here we demonstrate that treating the CEP with matrix metalloproteinase-8 (MMP-8) reduces the matrix constituents that impede solute uptake and thereby improves nutrient diffusion. Human CEP tissues harvested from four fresh cadaveric lumbar spines (age range: 38–66 years old) were treated with MMP-8. Treatment caused a dose-dependent reduction in sGAG, localized reductions to the amount of collagen, and alterations to collagen structure. These matrix modifications corresponded with 16–24% increases in the uptake of a small solute (376 Da). Interestingly, the effects of MMP-8 treatment depended on the extent of non-enzymatic glycation: treated CEPs with high concentrations of advanced glycation end products (AGEs) exhibited the lowest uptake compared to treated CEPs with low concentrations of AGEs. Moreover, AGE concentrations were donor-specific, and the donor tissues with the highest AGE concentrations appeared to have lower uptake than would be expected based on the initial amounts of collagen and sGAG. Finally, increasing solute uptake in the CEP improved cell viability inside diffusion chambers, which supports the nutritional relevance of enhancing the transport properties of the CEP. Taken together, our results provide new insights and *in vitro* proof-of-concept for a treatment approach that could improve disc nutrition for biologic therapy: specifically, matrix reduction by MMP-8 can enhance solute uptake and nutrient diffusion through the CEP, and AGE concentration appears to be an important, patient-specific factor that influences the efficacy of this approach.

## Introduction

Low back pain is the most common and most costly musculoskeletal condition [1], and is significantly associated with intervertebral disc degeneration [2]. Current medical interventions for disc degeneration are surgical in nature and are often unsuccessful, which motivates development of noninvasive alternatives. Noninvasive treatments to regenerate the disc and alleviate pain are largely experimental and focus on implanting new cells to produce matrix lost during degeneration [3–5], or injecting growth factors [6], genes [7], or other small molecules [8, 9] to stimulate matrix synthesis or reduce catabolism and inflammation. Importantly, all of these biologic therapies require a rich nutrient supply to sustain higher cell numbers or metabolic rates. However, the avascular and degenerated disc has a poor nutrient supply [10], which may limit the utility of biologic therapies [11–13]. Development of treatment strategies to improve disc nutrition may therefore expand the application and utility of biologic therapy as well as inform alternative approaches for slowing or reversing degeneration.

Proper disc nutrition involves nutrient and metabolite exchange between the nucleus pulposus (NP) cells and vertebral capillaries, and several factors can impair the normal patterns of solute exchange. For example, nutrients entering the disc and exiting metabolites must diffuse through the cartilage endplates (CEP), and diffusion could be hindered by age- or degeneration-related changes to the CEP matrix, including dehydration [14], mineralization [15–17], and fibrosis [14, 17, 18]. In addition to altering the mechanical functionality of the CEP [19], which is also physiologically important [20, 21], these matrix changes impact CEP biotransport functionality too. Specifically, dehydration prevents solutes from diffusing freely within the CEP; and increased deposition of proteoglycan, collagen, and mineral limits the amount of pore space available to solutes [22, 23]. Indeed, we recently found that CEPs with higher amounts of collagen, aggrecan, and mineral hindered nutrient diffusion, thereby impairing NP cell survival and function [17]. This suggests that discs with deficient CEP transport properties may be poor candidates for biologic therapies that increase nutrient demands; it also motivates strategies for enhancing solute transport through the CEP to improve nutrition and cell survival.

Strategies for enhancing solute transport through the CEP are underexplored. Since solute-hindering CEPs had higher amounts of collagen and aggrecan [17], one potential strategy involves matrix modification to reduce collagen and aggrecan. To this end, several human and bacterial enzymes have activity for these matrix constituents, including collagenases, aggrecanases, and gelatinases. Human matrix metalloproteinases (MMPs) are attractive candidates since they are naturally expressed in the intervertebral disc [24, 25]. Among MMPs, MMP-8 has selectivity for type II collagen (the main collagen present in the CEP [26]) and aggrecan. Thus, we sought to test the concept of matrix modification for enhancing CEP transport properties by enzymatic removal of matrix constituents using human MMP-8. Although MMP-8 has been used to enhance the transport properties of tumor tissues [27, 28], its effects on cartilage are unknown. We hypothesized that MMP-8 would liberate collagen and aggrecan from the CEP matrix and enhance CEP transport properties.

## Materials and methods

### Instrumentation

Fluorescence or absorbance measurements were performed on a SpectraMax M5 microplate reader (Molecular Devices, San Jose, CA) or on a FluoroLog-3 spectrofluorimeter (Horiba Jobin Yvon, Kyoto, Japan) with data collection using SoftMaxPro (Molecular Devices) or FluorEssence (Horiba Jobin Yvon), respectively. High-performance liquid chromatography (HPLC) was performed on an 1100 HPLC from Agilent (Santa Clara, CA). Fourier transform infrared spectroscopy (FTIR) imaging was performed on a Spotlight 400 imaging system from Perkin Elmer (Waltham, MA). Cryo-sectioning was done using a Microm HM550 cryostat (Thermo Fisher Scientific, Waltham, MA). Particle size measurements were carried out using a Malvern Nano-ZS Dynamic Light Scattering Instrument (Malvern Panalytical, Westborough, MA). Fluorescence microscopy was performed using MZ FLIII and DMi8 microscopes (Leica Microsystems, Wetzlar, Germany).

### Materials

Terrific Broth (TB), and isopropyl β-D-1-thiogalactopyranoside (IPTG) were purchased from VWR (Radnor, PA). Nickel Sepharose high performance resin prepacked in 5 mL HiTrap columns (HisTrap FF) and a Superdex 75 size exclusion chromatography (SEC) column were purchased from GEHealthcare (Piscataway, NJ). EDTA-free protease inhibitor solution was purchased from BiMake (Houston, TX). Amicon spin filters were purchased from Millipore (Billerica, MA). MMP-8 antibody (PA5-28246) and Pierce ECL Western Blotting Substrate were purchased from Thermo Fisher. SDS and zymogram materials and buffers were purchased from BioRad (Hercules, CA). Enzymatic activity was assayed using an EnzChek Gelatinase/Collagenase Assay Kit (E12055) purchased from Life Technologies (Carlsbad, CA). BL1(DE3) competent cells, Gibson Assembly Mastermix, and T4 DNA ligase were purchased from New England Biolabs (Ipswich, MA). Herculase II Fusion DNA polymerase was purchased from Agilent. MMP-8 (30915305) cDNA was purchased from GE Dharmacon (Lafayette, CO). AccQ-Tag derivatization kit (186003836) was purchased from Waters (Milford, MA). All lipids were purchased from Avanti Polar Lipids (Alabaster, AL), and lipid fluorophore 1,1′-dioctadecyl-3,3,3′,3′- tetramethylindodicarbocyanine, 4-chlorobenzenesulfonate salt (DiD; 60014) was purchased from Biotium (Fremont, CA). Barium fluoride windows for FTIR were purchased from Edmund Optics (Barrington, NJ). Cell viability was assessed using the Invitrogen Cytotoxicity assay from Thermo Fisher Scientific. Cartilage endplates were acquired from four fresh cadaveric lumbar spines obtained from donors with no history of musculoskeletal disorders (UCSF Willed Body Program). All other reagents were purchased from Sigma–Aldrich (St. Louis, MO).

### Plasmid construction

Plasmids were cloned using standard techniques, including Gibson assembly with sequence verification. MMP-8 (BC074989) plasmids were constructed using the primers in S1 Table.

### MMP-8 cloning

Truncated MMP-8 (M100-G262) for periplasmic expression was cloned from MMP-8 cDNA (BC074989) using primer set 1 by Gibson cloning (S1 Table). Primers were designed for insertion of MMP-8 into a pET22B vector with a C-terminal poly-histidine tag [29]. All PCR reactions were performed with Herculase II Fusion DNA polymerase. Following PCR, amplification products were gel-purified and combined following the Gibson master mix protocol. All plasmids were confirmed by DNA sequencing. After unsuccessful expression from the periplasmic space, the PelB header was removed and a GGS spacer was placed between the enzyme and the poly-histidine tag to allow for MMP-8 expression from inclusion bodies using primer sets 2 and 3. Primers were phosphorylated to allow for self-ligation following PCR reactions. All PCR reactions were performed with Herculase II Fusion DNA polymerase. Following PCR amplification products were gel purified and ligated using T4 DNA ligase. All plasmids were confirmed by DNA sequencing.

### Protein expression and purification

pET22b-MMP-8-GGS-His_6_ were transformed into BL21-Codon Plus (DE3)-RIPL *E*. *Coli* cells and grown overnight on ampicillin plates. A streak of colonies was transferred into terrific broth, grown overnight, and inoculated at 1% into a larger culture containing 100 mg/mL ampicillin. Cultures were allowed to grow for 18 h with an induction using 0.5 mM IPTG at A_280_ = 0.7–1.0. Cells were centrifuged at 10,000 x*g* for 30 min at 4°C. The pellet was resuspended in lysis buffer (100 mM Tris, 5 mM CaCl_2_, 0.5 mM ZnOAc, 0.05% Brij-35, pH 7.5, EDTA-free protease inhibitor) and lysed cells using four freeze–thaw cycles followed by tip sonication at 12W for 3x10 s, alternating with ice to keep the suspension chilled. The solution was pelleted by centrifugation at 30,000 x*g* for 30 min at 4°C. The supernatant was removed, and the insoluble pellet was resuspended in 20 mL of lysis buffer (with protease inhibitor) by vigorously pipetting to a homogeneous suspension then pelleted by centrifugation at 30,000 x*g* at 4°C. The process was repeated once more to wash the pellet with 20 mL MilliQ water containing protease inhibitor. The washed pellet containing the bacterial inclusion bodies was resuspended at 4°C using extraction buffer (20 mM Tris–HCl, 500 mM NaCl, 10% glycerol, 8 M urea, pH 8.0) and allowed to solubilize for 1–2 h at room temperature on an orbital shaker. The extraction mixture was centrifuged at 30,000 x*g* for 30 min and the supernatant was filtered through a 0.45 μm filter in preparation of Ni^2+^ affinity chromatography.

### Protein purification and refolding

MMP-8 was purified using a modified column refolding protocol [30]. Four joined 5 mL HisTrap FF columns charged with Ni^2+^ were equilibrated with extraction buffer containing 20 mM imidazole. The clarified supernatant was loaded equally onto each column then joined and washed with extraction buffer for 10 column volumes (CV). Samples were taken through a stepwise titration with 10 CV at 8 M, 6 M, 4 M, 2 M, 1 M, and 0 M Urea by combining extraction buffer with refolding buffer (20 mM Tris–HCl pH 8.0, 500 mM NaCl, 10% glycerol, 0.5 mM oxidized glutathione, 5 mM reduced glutathione) at desired ratios. For example, 8 M Urea = 100% extraction buffer and 6 M Urea = 75% extraction buffer, 25% refolding buffer. Bound protein was eluted with refolding buffer containing 400 mM imidazole. Fractions were analyzed by SDS-page, pooled, and then dialyzed against 20 mM Tris–HCl, 50 mM NaCl, 0.1 mM DTT before purification by size-exclusion chromatography.

### Size-exclusion chromatography

Dialyzed samples were concentrated using Amicon 10 kDa MWCO spin filters before purification by size exclusion chromatography. Briefly, a Dionex FPLC equipped with a Superdex 75 column was operated at a flow rate of 0.5 mL/min in 20 mM Tris–HCl, 50 mM NaCl, 0.1 mM DTT and the eluate was monitored at 280 nm. Samples were analyzed by SDS-Page with like fractions combined.

### Enzyme activity

Enzyme activity was determined using the EnzChek Gelatinase/Collagenase Assay Kit following manufacturer recommendations using the included gelatin fluorophore. Activity was calibrated using a known amount of *Clostridium* collagenase supplied with the kit. One unit is defined as the amount of enzyme required to liberate 1 μmole of L-leucine equivalents from collagen in 5 h at 37°C.

### Western blot

Western blot was performed on MMP-8 using standard techniques. Proteins were loaded on a 4–20% SDS-PAGE Gel and transferred onto a blotting membrane. The blotting membrane was incubated with blocking buffer (20 mM Tris, 140 mM NaCl, 5% Milk, 0.1% Tween, 1% PMSF, pH 7.5), washed, and mixed overnight with 1:5000 Rabbit Anti-MMP-8 (Thermo Fisher, PA5-28246) at 4°C. The membrane was thoroughly washed before addition of the secondary antibody (1:10000 anti-rabbit HRP). Following incubation for 1 h, the membrane was washed before addition of Pierce ECL Western Blotting Substrate. The membrane was then imaged using a film development cassette.

### Zymography

Gelatin zymography was performed following BioRad protocols. Protein sample was mixed 1:1 with zymogram loading buffer, then loaded onto a zymography gel and run in 1X Tris-Glycine containing 0.1% SDS. After 90 min at 100 V the gel was extracted and washed thoroughly with water before being placed in renaturation solution for 30 min. Gels were transferred into Development Solution at 37°C overnight and subsequently stained with Coomassie Brilliant-Blue R-250 for 1 h at room temperature. Loss of gelatin was visualized by development with destaining solution for 1 h until clear bands appeared.

### Liposome formation

Fluorescently labeled liposomes were used to evaluate large-solute uptake and were made using a thin-film method. Dry lipid films containing 1,2-distearoyl-*sn*-glycero-3-phosphocholine (DSPC),1,2-distearoyl-glycero-3phosphoethanolamine-N-(polyethyleneglycol)-2000 (DSPE-PEG2K), DiD at 55:40:05:0.02 mole ratio were rehydrated in 20 mM HEPES, 140 mM NaCL, pH 7.4. Each sample was heated at 60°C for 1 h and then sonicated at 60°C for 10 min. Liposomes were then extruded 11–13 times through a 100 nm polycarbonate membrane with subsequent verification of the particle size using light scattering on a Malvern Zetasizer.

### Cartilage endplate treatment

Intact human cartilage endplates were harvested from four fresh cadaveric lumbar spines (age range: 38–66 years old; mean age: 56 ± 10 years) belonging to donors with no history of musculoskeletal disorders or disc herniation (S2 Table). This study involving cadaveric tissues was exempt from institutional approval. From each L4 and L5 disc, full-thickness CEP samples including any calcified cartilage, were removed from the subchondral bone adjacent to the nucleus pulposus with a razor blade. The CEPs derived from the L4 and L5 discs were treated as independent samples because there was no pairwise association or agreement between the transport properties of the L4 and L5 CEP tissues. From these CEPs, circular biopsies (4 mm diameter) were prepared and bisected perpendicular to the CEP surface to create two semi-circular halves: one half for treatment and the other for site-matched control. CEP samples were placed in 100 μL of collagenase reaction buffer (50 mM Tris-HCl, 150 mM NaCl, 5 mM CaCl_2_, 0.2 mM sodium azide, pH 7.6) with or without 0.2 U/mL, 2 U/mL, or 20 U/mL recombinant MMP-8 (*n* = 8 site-matched half biopsies per group, comprised of 2 half biopsies per donor from all 4 donors). Samples were placed on an orbital shaker and mixed overnight for 18 h at 37°C. The digest supernatant was removed and stored for subsequent biochemical analysis. CEP samples were washed 3X with PBS, blotted dry, and transferred to microcentrifuge tubes containing 200 μL of 0.1 mg/mL sodium fluorescein (376 Da) or 2 mM liposomes. After equilibrating overnight in the fluorescein solution, the CEP samples were extracted, blotted dry, weighed, and dehydrated by lyophilization at 80°C for 2 h. The dehydrated samples were re-weighed and dissolved in 200 μL of 1 mg/mL papain at 60°C overnight. Papain digests were centrifuged at 2,000 x*g* for 20 min, and the supernatant was assayed for protein and fluorophore contents.

### Solute uptake

Papain digests were diluted in PBS, and fluorophore concentration was determined by fluorescence emission (fluorescein: ex. 450 nm, em. 516 nm; DiD: ex. 644 nm, em. 663 nm). Fluorescence intensities were referenced to a standard curve of known concentrations. Percent uptake was computed by dividing the mass of solute in the tissue by the total mass of solute added to the equilibration solution. Since the sample preparation procedures resulted in biopsy halves with inconsistent sizes, and since the mass of the biopsy half was positively correlated with percent uptake, the percent uptake values were normalized to the mass of the CEP biopsy half. Paired *t*-tests were used to compare solute uptake between site-matched control and treated halves; *p* < 0.05 (2-tailed) was considered statistically significant.

### Proteoglycan content

Sulfated glycosaminoglycan (sGAG) content was measured using a dimethylmethylene blue assay [31]. Percent sGAG released from the CEP was computed by dividing the sGAG in the reaction digests by the initial sGAG, which was determined by summing the sGAG from both the reaction and papain digests. Fixed charge density was estimated assuming two moles of charge per mole of sGAG and a molecular weight of 502.5 g/mole sGAG [32]. Data were analyzed by one-way ANOVA with Tukey’s post-hoc tests; *p* < 0.05 (2-tailed) was considered statistically significant.

### Collagen content

Collagen content was determined by quantifying hydroxyproline from neutralized hydrolysates. Aliquots of papain digest and reaction digest were hydrolyzed in 6 N HCL for 18 h at 110°C. Acid hydrolysates were neutralized with sodium hydroxide and derivatized using Waters AccQ-Tag Derivatization kit following manufacturer directions. Samples were analyzed by HPLC on a C8 column with a gradient of 0–15% of MeOH with 0.1% TFA in H_2_O with 0.1% TFA. Hydroxyproline concentration was determined by calculating the peak area compared to a standard curve. Collagen content was calculated from the amount of hydroxyproline, assuming the latter accounts for 13.5% of the total collagen. The initial collagen content of the samples was determined by summing the collagen in the tissue post-treatment with the collagen in the reaction digests. A *t*-test was used to compare solute uptake between samples with the highest versus lowest collagen concentrations, and one-way ANOVA with Tukey’s post-hoc tests was used to test for donor differences. *p* < 0.05 (2-tailed) was considered statistically significant.

### Advanced glycation end products

The total concentration of advanced glycation end products (AGEs) was determined using a fluorimetric assay. Fluorescence readings of the neutralized lysates (ex. 370 nm, em. 440 nm) were referenced to a quinine sulfate standard [33] and then normalized to collagen content. Initial AGE content was computed by summing the AGEs remaining in the tissue with AGEs in the reaction digest. A *t*-test was used to compare solute uptake values between samples with the highest versus lowest AGE concentrations, and one-way ANOVA with Tukey’s post-hoc tests was used to test for donor differences. *p* < 0.05 (2-tailed) was considered statistically significant.

### FTIR imaging

Following overnight treatment, CEP samples were flash-frozen in Optimal Cutting Temperature (OCT) compound. Next, 7 μm-thick cryo-sections were placed on barium fluoride windows and imaged using an FTIR microscope. Images were acquired in transmittance mode with a 4 cm^-1^ spectral resolution and a 6.25 μm pixel size. Spatial maps of collagen (1595–1710 cm^-1^ Amide I peak area), aggrecan (960–1185 cm^-1^ carbohydrate peak area), and collagen order (ratio of collagen’s 1338 cm^-1^ CH_2_ side chain vibration peak area to collagen’s 1480–1590 cm^-1^ Amide II peak area) were acquired in 0.8 mm x 0.2 mm regions of interest (one ROI/section). The 1338 cm^-1^:Amide II ratio is sensitive to enzyme-induced degradation of the structural order of the collagen triple helix [34]. Spectral indices at normalized depths from the NP/CEP interface were calculated in control and treated CEPs (*n* = 3 CEPs/group, three adjacent sections/CEP). Calculations were performed in IDL 8.6 (Harris Geospatial Solutions, Broomfield, CO).

To describe the spatial fluctuations in CEP composition, we used regression models of the FTIR indices as a function of normalized depth from the NP/CEP interface, a continuous independent variable. For each of the indices, a polynomial model for normalized depth was fitted with random intercepts and slopes [35]: Amide I peak area, a 3^rd^-degree polynomial; carbohydrate peak area, a 2^nd^-degree polynomial model; and 1338 cm^-1^:Amide II ratio, a 4^th^-degree polynomial. We tested whether overall trajectories differed by treatment status via a post-estimation test using a contrast statement (F-test). Analyses were performed using SAS v 9.4; *p* < 0.05 (2-tailed) was considered statistically significant.

### Nucleus pulposus cell isolation

Nucleus pulposus (NP) cells were isolated from coccygeal discs obtained from steers (24–28 months old) collected at slaughter from a local abattoir (Marin Sun Farms, Petaluma, CA). The nucleus pulposus was removed from each disc, washed with sterile PBS containing 2% penicillin-streptomycin, and cut into approximately 0.125 cm^3^-sized pieces. The dissected tissue was digested inside 50 mL conical tubes containing standard cell growth medium supplemented with 0.8 mg/mL collagenase P (cat. no. 11 213 857 001). The standard growth medium was comprised of low-glucose Dulbecco’s modified Eagle’s medium (DMEM), 1% non-essential amino acids (Thermo Fisher), 5% fetal bovine serum (HyClone; Thermo Fisher Scientific), 2% penicillin-streptomycin, and 1.5% osmolarity salts (5 M NaCl and 0.4 M KCl). Samples were digested for 8 h at 37°C under constant agitation, run through 40 μm filters, and then centrifuged at 200 x*g* for 6 min. Supernatant was removed, and the pelleted NP cells were re-suspended in growth medium and expanded to passage 2 in 21%/5% O_2_/CO_2_ conditions. For the diffusion chamber experiments, the expanded NP cells were suspended in growth medium and mixed with low gelling temperature agarose (type VII, A4018) to give a final concentration of 1% agarose and 4 million cells/mL, which is the average nucleus pulposus cell density in the adult disc [36].

### Diffusion chambers

To determine if MMP-8 treatment improves nutrient diffusion through the CEP, we used diffusion chambers [17], which mimic the nutrient environment of the disc *in vivo*. Briefly, NP cells cultured inside the chamber obtain nutrients that diffuse through full-thickness CEP tissues at the open sides of the chamber (S1 Fig). By imposing culture conditions with identical chamber cell density (dictates nutrient demand), we determined how differences in transport properties between untreated versus treated CEPs affected NP cell viability. The design of the diffusion chambers was modified from a previous study [37]. Each chamber was comprised of two parallel glass slides (25 x 75 mm) separated by 170 μm-tall impermeable spacers. After overnight treatment, OCT-embedded CEP samples were cryo-sectioned perpendicular to the CEP surface (180 μm section width). CEP sections were then washed for 90 min with sterile PBS containing antibiotics: 0.012% penicillin, 0.02% streptomycin, and 0.01% gentamicin. Next, two coronally adjacent sections from each CEP were placed with the deep layer of the CEP at the open sides of the chambers (*n* = 12 sections per group, with 4 each from donors 1–3). Next, the NP cell-agarose mixture was pipetted into the center of the chambers, the chambers were placed in 100 mm-diameter dishes with 25 mL of growth medium, and the dishes were incubated for 72 h at 37°C. Owing to the mismatch between the CEP section thickness and the height of the spacers, the CEPs were exposed to a small compressive strain during incubation.

### Cell viability

Cell viability in the diffusion chambers at the end of the incubation period was assessed using a cytotoxicity assay. After removing the chambers from the incubator, the agarose gels were rinsed with 600 μL of PBS, covered with 200 μL of PBS containing 1 μL/mL calcein-AM and 4 μL/mL ethidium homodimer-1, and incubated for 30 min at 37°C. After incubation, the stained gels were rinsed with 400 μL PBS and imaged using fluorescence microscopy. Low magnification images (MZ FLIII; 1x objective) of each half of the gel were acquired for semi-quantitative analysis of viable distance using the ruler tool in ImageJ (NIH, USA). For quantitative analysis of viable cell percentages across the chambers, higher magnification images (DMi8; 5x Plan Apo objective) were acquired using the automated stage controls for scanning, imaging, and tiling. Viable cell percentages were calculated using the cell counting tool in ImageJ. Viable distance was defined using the 90% viability criterion and was averaged at five locations per chamber (2 chambers per donor for donors 1–3). Semi-quantitative estimates of viable distance were within 10% of measurements derived from the high magnification analysis. Paired *t*-tests were used to compare viable distance between chambers with control and treated CEP tissues from the same endplate; *p* < 0.05 (2-tailed) was considered statistically significant.

## Results

### MMP-8 purification & activity

**Fig 1:**
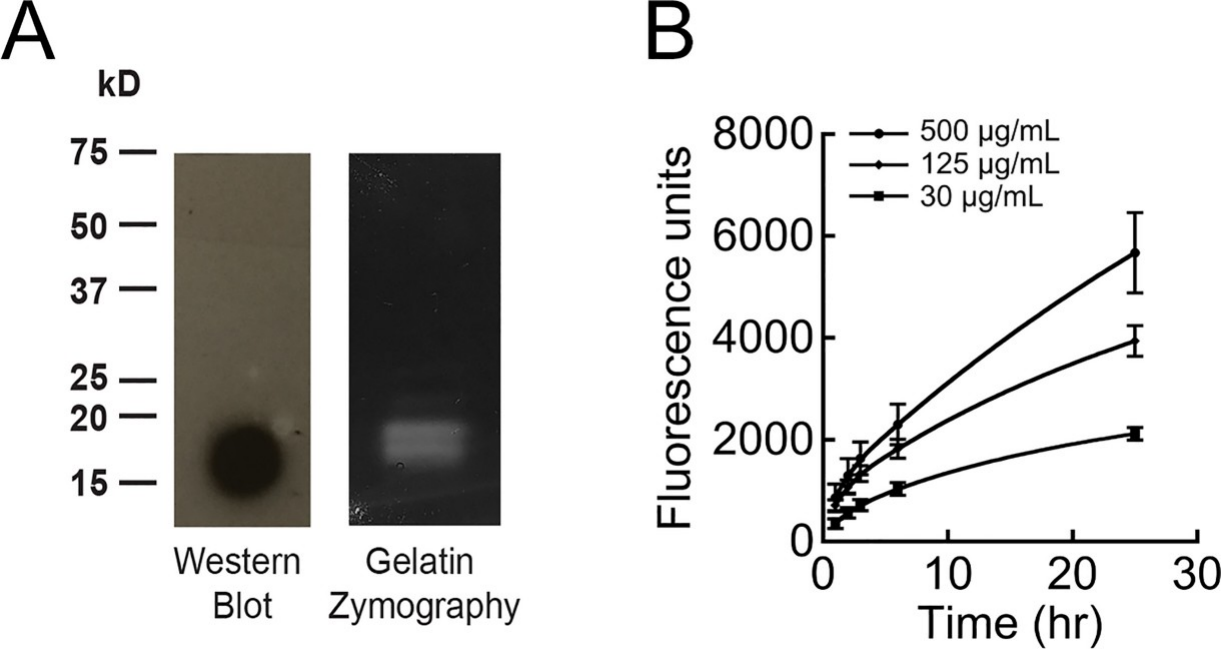
Purification and characterization of MMP-8. MMP-8 was purified using a column refolding protocol. (A) Enzyme mass was confirmed by Western blot (left) and gelatin zymography (right). (B) Purified protein displayed dose-dependent activity for fluorescently labeled gelatin.

Active truncated (M100-G262) MMP-8 was recombinantly expressed from *E*. *coli*. Recombinant protein mass of approximately 19 kDa was confirmed by Western blot (Fig 1A), and purity was confirmed by Coomassie gel (S2 Fig). To determine if the enzyme was active against matrix components, activity was first evaluated by gelatin zymography, which revealed gelatin removal at the expected 19 kDa (Fig 1A). In addition, dose-dependent activity over a 24 h period was measured using a gelatin fluorophore, where fluorescence increased with cleavage (Fig 1B).

### MMP-8 activity on CEP matrix composition

**Fig 2:**
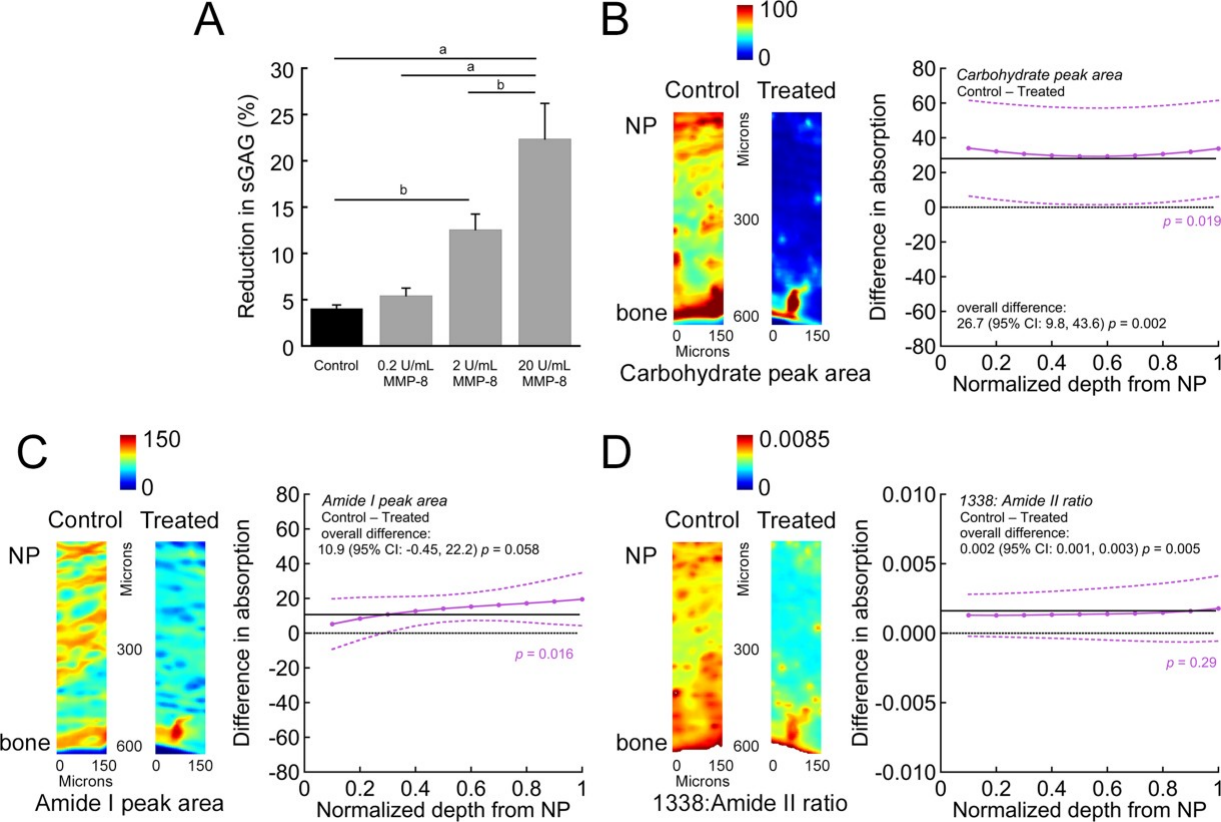
MMP-8 treatment liberates extracellular matrix components from the CEP. CEP samples were treated for 18 h with MMP-8. (A) Treatment caused the dose-dependent removal of sGAG, evaluated by DMMB assay. Bars show mean ± SEM. One-way ANOVA (*p* < 0.0001) with Tukey’s post-hoc test, ^a^ *p* < 0.001, ^b^ *p* < 0.01. *n* = 8 CEPs per group, comprising two each from donors 1–4. (B)-(D) Representative FTIR absorption maps and spatial plots of the mean difference in absorption between control and MMP-8-treated CEP tissues for (B) carbohydrate peak area (960–1185 cm^-1^, estimate of aggrecan content), (C) Amide I peak area (1595–1710 cm^-1^, estimate of collagen content), and (D) 1338 cm^-1^: Amide II peak ratio (estimate of collagen structural order). Tissues for FTIR imaging were treated with 20 U/mL MMP-8. Spatial plots show the mean difference in average absorption (purple, with 95% confidence intervals from *n* = 3 CEPs/group and 3 sections/CEP) as a function of normalized depth from the NP/CEP interface between control *vs*. MMP-8-treated CEPs (control minus treated; > zero suggested higher absorption in the control CEPs). Solid black lines indicate overall difference, *i*.*e*. for all depths pooled, and dotted black lines indicate no difference. *p*-values in purple indicate statistical significance of the difference between control and treated CEPs as a function of depth, and *p*-values in black indicate statistical significance of the overall difference. Overall, compared to the control CEPs, the treated CEPs had lower carbohydrate peak area (B), Amide I peak area (C), and collagen order (D).

MMP-8 liberated matrix from human CEP tissues. MMP-8 displayed a dose-dependent reduction in total sGAG from the CEP (Fig 2A), with the highest dose releasing over 20% of sGAG. These reductions in sGAG coincided with a decrease in computed fixed-charge density (S3 Fig). FTIR imaging of CEP sections corroborated the bulk reductions in sGAG, with sections from treated samples showing significantly lower carbohydrate peak area (Fig 2B). MMP-8 treatment also reduced the amount and structural order of the collagen. Specifically, FTIR imaging revealed that treated samples had lower Amide I peak area, particularly in the deeper zones of the CEP (Fig 2C). To determine if enzymatic treatment had any broader effects on the collagenous matrix, we measured the 1338 cm^-1^: Amide II peak ratio, which is a measure of collagen structural order that is sensitive to collagenase activity [34]. MMP-8 treated samples had significantly lower ratios overall, indicating decreased structural order of the triple helix and increased denaturation (Fig 2D). Bulk measures of total collagen by hydroxyproline and total AGE concentration by fluorescence assay were insensitive to MMP-8 treatment (*p* > 0.05).

### MMP-8 activity on solute uptake in the CEP

**Fig 3:**
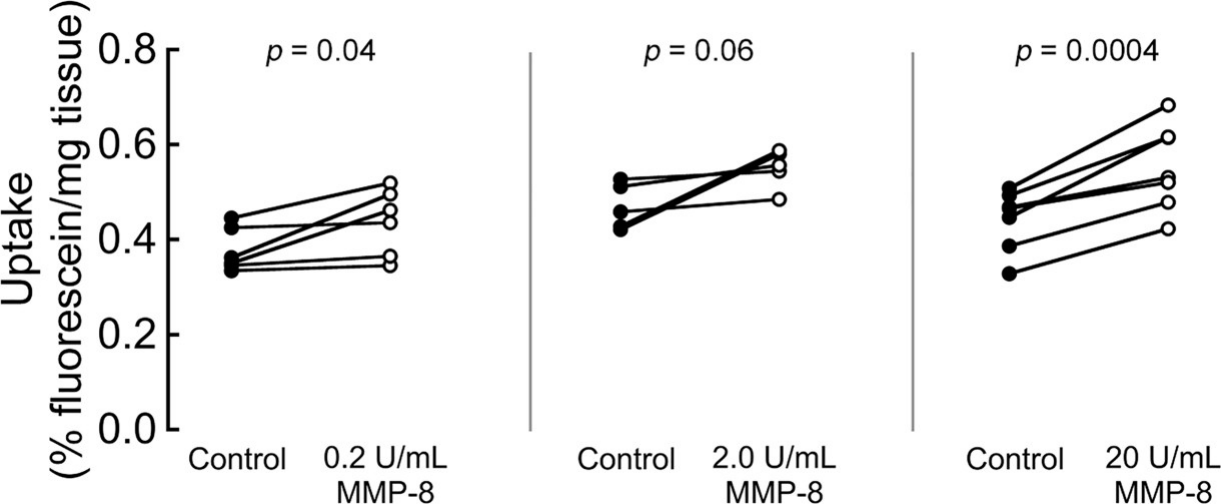
MMP-8 improves sodium fluorescein uptake in CEP tissues. CEP samples treated for 18 h with 0.2 (left), 2 (middle) or 20 (right) U/mL of MMP-8 show increased percent uptake of sodium fluorescein. Each pair represents site-matched matched biopsy halves. n = 8 CEP samples per group, comprising two each from donors 1–4.

To resolve if the matrix modifications caused by MMP-8 treatment enhanced the transport properties of the CEP, we measured the uptake of sodium fluorescein (376 Da). MMP-8 treatment significantly increased fluorescein uptake (Fig 3): site-matched samples showed average increases in uptake (mean ± SEM) of 16 ± 6%, 19 ± 8%, and 24 ± 4% with 0.2 U/mL, 2 U/mL, and 20 U/mL MMP-8, respectively. Treatment with 0.2 U/mL MMP-8 also increased the uptake of large liposomal nanoparticles (~800 kDa, 100 nm diameter) by 561% (S4 Fig). Owing to the large size of the liposomes, the total percent uptake of liposomes was much lower than sodium fluorescein, which may partly explain the greater percent increase with MMP-8 treatment.

### Determinants of solute uptake with MMP-8 treatment

**Fig 4:**
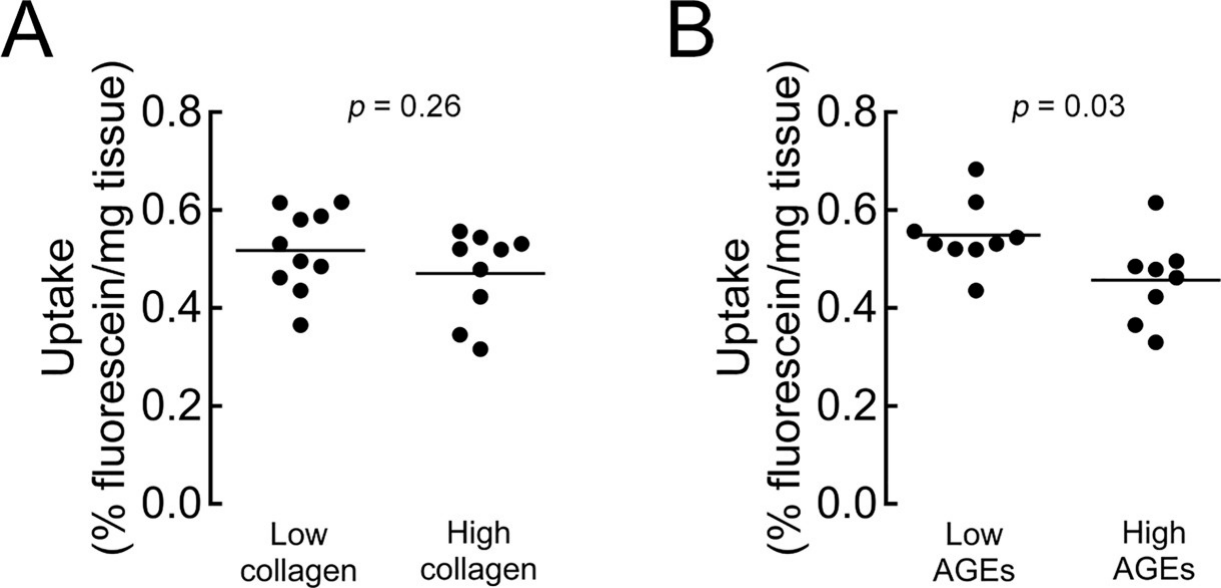
Collagen cross-linking restricts sodium fluorescein uptake in treated CEP tissues. (A) Treated samples across all doses exhibited similar uptake regardless of collagen content (high: >700 μg/mg dry weight; *t*-test, *p* = 0.26). (B) Treated samples across all doses exhibited lower uptake in the samples with high AGE concentration (high: >0.75 ng quinine fl/μg collagen; *t*-test, *p* = 0.03). Each symbol represents a biopsy half from either the L4 or L5 disc of one of four donor spines studied.

**Fig 5:**
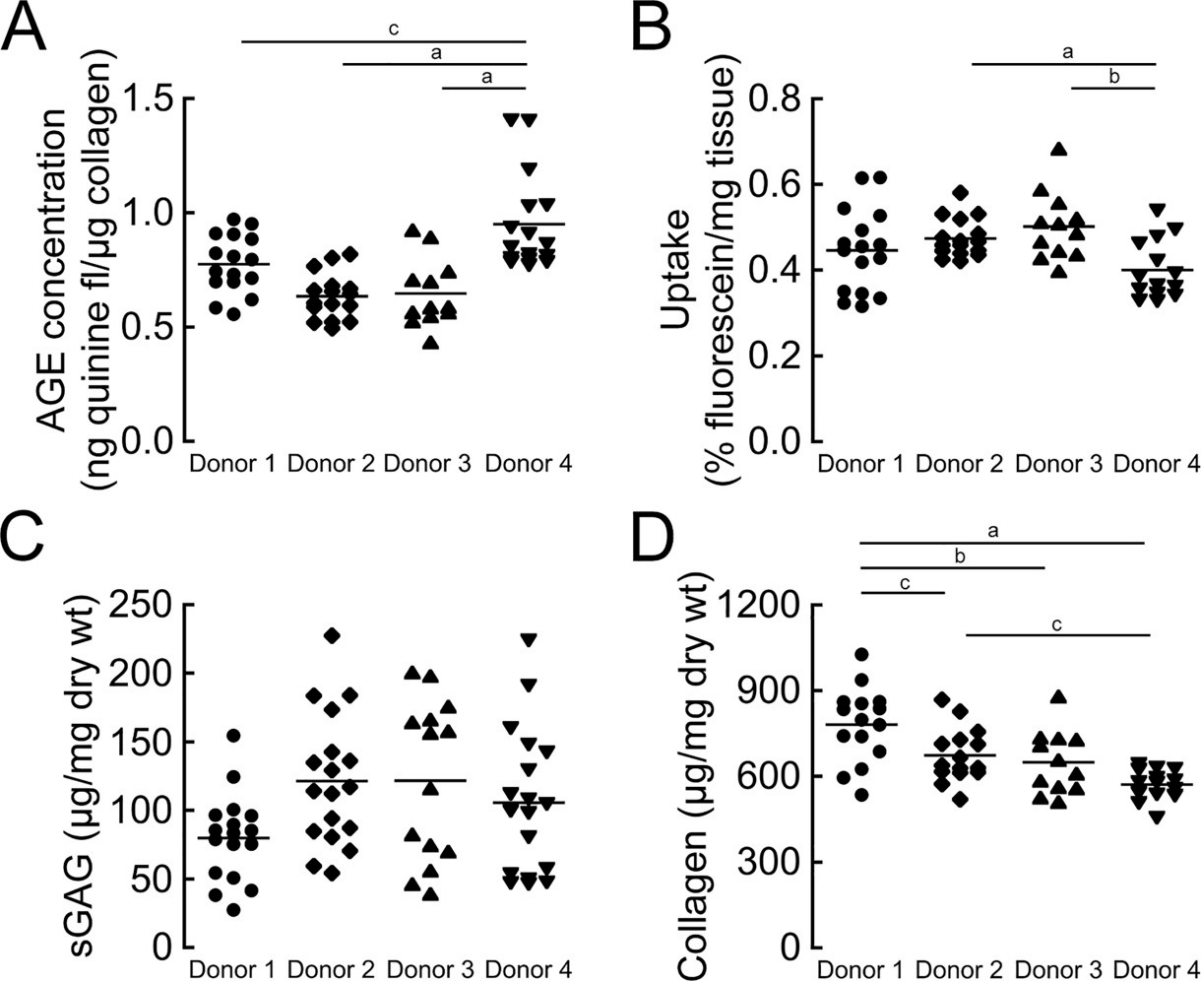
CEP donors exhibit varying levels of ECM components and transport properties. (A) Comparison of initial AGE content between donors. Donor 1 and donor 4 had CEP tissues with elevated AGE concentrations (One-way ANOVA, *p* = 0.03). (B) Fluorescein uptake was lowest in CEP tissues from donor 4, which had the highest AGE concentrations (One-way ANOVA, *p* = 0.04). (C) Initial sGAG content was similar in CEP tissues from all donors (One-way ANOVA, *p* = 0.05). (D) Initial collagen content was lowest in donor 4 (One-way ANOVA, *p* = 0.02). Each symbol represents a biopsy half; all samples pooled (treated and untreated). Tukey’s post-hoc test, ^a^*p* < 0.001, ^b^*p* < 0.01, ^c^*p* < 0.05.

To understand the factors that influence solute uptake following MMP-8 treatment, we investigated the roles of collagen quantity and quality. Separating the MMP-8-treated samples into equal-sized groups with low versus high collagen contents hinted that samples with higher collagen contents pre-treatment had lower fluorescein uptake (Fig 4A), although the difference was not statistically significant (*p* = 0.26). Instead, uptake post-treatment was more strongly related to the degree of non-enzymatic glycation: the treated CEP samples with the lowest AGE concentrations pre-treatment had 20% greater fluorescein uptake on average (*p* = 0.03) compared to treated samples with the highest AGE concentrations pre-treatment (Fig 4B). AGE concentrations affected fluorescein uptake in the untreated CEP tissues too: CEP tissues with high AGE concentrations had 19% lower uptake on average (*p* = 0.0003, S5 Fig). Moreover, AGE concentrations and treatment effects were donor-specific. CEP tissues from donor 4, and to a lesser extent donor 1, had higher AGE concentrations compared to the others (Fig 5A), and the elevated AGE concentrations in the CEP tissues from those donors coincided with lower uptake (Fig 5B). This was true despite donor 4 having similar sGAG (Fig 5C) and less collagen (Fig 5D) initially, which would be expected to associate with greater uptake [22].

### MMP-8 activity on nutrient transport in the CEP

**Fig 6:**
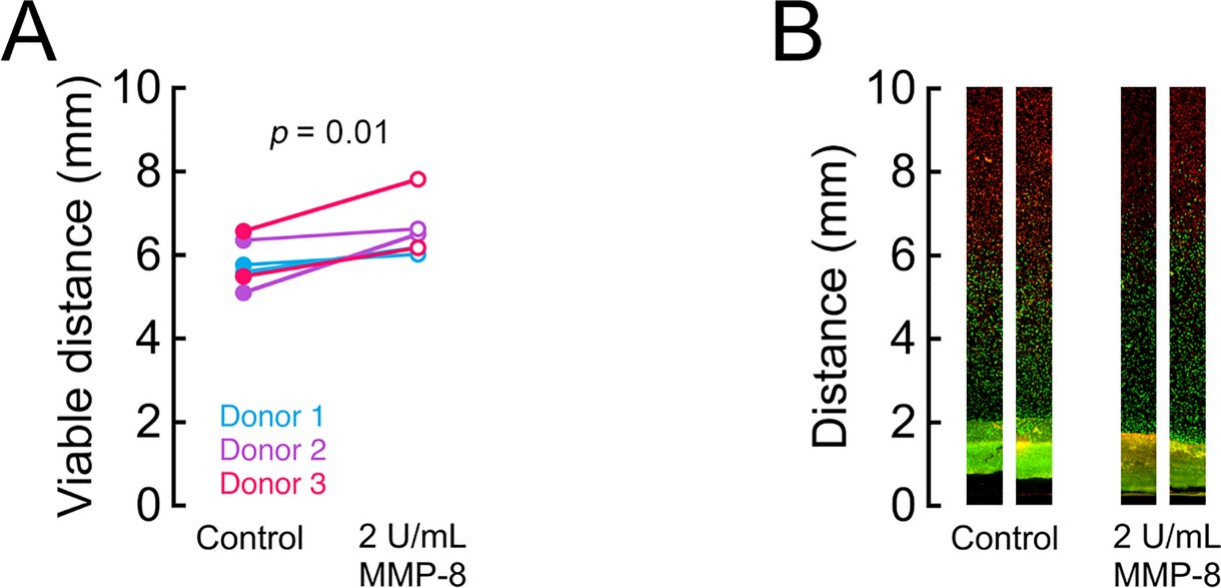
MMP-8 improves nutrient diffusion in CEP tissues. (A) Diffusion chambers containing CEP samples treated for 18 h with 2 U/mL of MMP-8 showed increased viable distance, indicating greater glucose availability. Mean difference: 0.74 mm; 95% CI: 0.23, 1.25, *p* = 0.01 (B) Representative photomicrographs of the diffusion chamber showing the transition between live (green) and dead cells (red). Distance was measured from the open side of the chamber. Chambers were cultured for 72 h with 4 million NP cells/mL.

To determine the biologic relevance of matrix modification with MMP-8 treatment, we tested the CEP tissues from donors 1–3 in diffusion chambers containing bovine NP cells. A single dose of 2 U/mL was chosen because this dose showed the greatest reduction in matrix with the smallest loss in fixed charge density. MMP-8 treatment at 2 U/mL significantly increased the viable distance in the diffusion chambers by an average 13% (range: 4–28%; Fig 6), indicating improved nutrient diffusion through the CEP between the culture medium outside the chambers and the NP cells inside the chambers.

## Discussion

These results show that MMP-8 treatment improves the uptake of a small solute into cadaveric human CEP tissues (*p* = 0.0004 to 0.06). This increase in uptake was mainly driven by a greater amount of pore space available to the solutes. For example, MMP-8 treatment led to dose-dependent reductions in sGAG in the CEP by up to 20%, which coincided with increases in the uptake of fluorescein by 16–24%. MMP-8 treatment also caused localized reductions to the amount of collagen and alterations to collagen structural order. Importantly, the effects of MMP-8 treatment depended significantly on the extent of non-enzymatic cross-linking of the CEP matrix: overall, treated CEP tissues with the lowest AGE concentrations showed 20% greater fluorescein uptake following treatment compared to treated tissues with the highest AGE concentrations. Moreover, AGE concentrations in the CEP were donor-specific, and the donor with CEP tissues having the highest AGE concentrations showed the lowest solute uptake, despite having a similar amount of sGAG initially and the lowest amount of collagen compared to the other donors. Finally, increasing solute uptake improved cell viability inside diffusion chambers, which supports the biologic relevance of enhancing the transport properties of the CEP and indicates increased nutrient diffusion. Poor nutrient diffusion through the CEP impairs disc nutrition [12, 17] and may limit the efficacy of regenerative therapy [13, 17]. Yet, we are aware of no treatment approaches for improving diffusion. Taken together, our results provide new insights and *in vitro* proof-of-concept into a treatment approach that could potentially improve nutrition for biologic therapy: specifically, matrix reduction by MMP-8 can enhance solute uptake and nutrient diffusion through the CEP, and AGE concentration appears to be an important, patient-specific factor that influences the efficacy of this approach.

Our results indicate that MMP-8 treatment may reverse some of the age- or degeneration-related factors that could hinder nutrient transport. For example, in our prior study we reported that CEPs that hindered nutrient diffusion had multiple compositional deficits consistent with fibrotic changes, including higher amounts of collagen and aggrecan, more mineral, and fewer mature cross-links [17]. Other groups have reported similar relationships between solute transport and the amounts of various matrix constituents, including collagen, aggrecan, and mineral [16, 22, 23, 36]. It is not presently clear which of these deficits is most important, or if reversing only some of the deficits is sufficient. Notwithstanding, MMP-8 treatment reduced the amounts of collagen and aggrecan and improved nutrient diffusion. Together, the available data suggest that fibrotic changes play a significant role in poor disc nutrition and that MMP-8 treatment has mechanistic effects.

Examination of the effects of MMP-8 treatment revealed a tradeoff between liberating matrix to increase pore space versus maintaining CEP swelling pressure. Cartilage swelling is a balance between the negative fixed charge density of the proteoglycans and tensile stresses in the collagen network, which resist tissue swelling [38]. Here, MMP-8 treatment induced damage to the collagen network and increased uptake, despite the loss in sGAG. This is consistent with results following collagenase treatment of articular cartilage [39]. However, raising the MMP-8 dose released increasing amounts of sGAG from the CEP, but the corresponding improvements in solute uptake did not scale with sGAG release (Figs 2 and 3). This suggests that although collagen damage in combination with some loss of sGAG improves solute uptake, removal of too much sGAG is counterproductive since it lowers fixed charge density (S2 Fig) and swelling pressure.

Removal of too much matrix from the CEP or inducing excessive collagen damage could also have biomechanical consequences. For example, the collagen network helps resist tissue deformation [19, 40], and proteoglycans in the CEP are believed to help prevent the loss of proteoglycan aggregates from the nucleus pulposus [22]. Additional work is required to determine if appreciable improvements in solute transport can be achieved without compromising CEP biomechanical function.

An important finding of this study is that solute uptake in both treated and untreated CEPs was lowest in tissues with high AGE concentrations (Fig 4, S5 Fig). AGEs are formed through non-enzymatic glycation of the free amino groups of proteins by reducing sugars, and AGE accumulation in low-turnover proteins of the disc increases with ageing [41] and is accelerated by metabolic disease [42–44] and with AGE-rich diets [45]. High AGE concentrations could negatively impact solute uptake in several ways. First, AGEs decrease water content by lowering the hydrophilic charge of the GAGs [46], which could reduce the volume of water available for solute diffusion. Second, increased inter- and intra-molecular crosslinks may hinder the release of degraded protein fragments, which could limit solute penetration. Although a larger sample size with greater variation in AGEs is needed to elucidate their role, these factors could explain why solute uptake in CEPs with high AGE concentrations appeared to be lower than expected based on the amounts of collagen and sGAG alone.

Related, our finding that solute uptake was lower in treated CEPs with high AGE concentrations is also consistent with known effects of AGEs on proteolysis. Specifically, AGEs can directly impair the matrix-digesting activity of MMPs by altering the structure of the protein and thereby interfering with enzyme-matrix interactions [47–49]. Altogether, these results suggest that application of matrix-modifying enzymes to improve nutrient transport may require tuning to patient-specific factors such as matrix cross-linking.

Although we focused on MMP-8, it is important to note that other enzymes with activity for CEP matrix constituents may achieve similar effects. It is also possible that a combination of matrix-modifying enzymes may allow for optimal removal of inhibitory matrix constituents while maintaining tissue integrity. These issues will be essential for future *in vivo* studies. Regardless of the specific enzyme selected, special considerations will be required when using native forms of the enzyme with post-translational modifications that can affect enzymatic activity and regulation.

This study had several limitations. First, we evaluated the effects of MMP-8 in diffusive conditions that occurred under a free-swelling environment, while static or dynamic compressive loading of the CEP could increase transport via forced solute convection [50]. The role of solute convection in disc nutrition is unclear, but we expect the present data to be representative of the relative effects of MMP-8.

A second limitation is that we treated CEP tissues *in vitro*, and treating CEP tissues *in vivo* warrants several considerations. Specifically, CEP tissues were denuded of cells, and studying CEP tissues with viable chondrocytes may be important in accounting for local nutrient gradients over time, or to address long-term changes in CEP matrix turnover. However, we presently sought to take a snapshot of the compositional characteristics of the CEP, and from that, identify how variations in those characteristics and their response to MMP-8 treatment impacts nutrient diffusion. In this context, we believe the lack of viable chondrocytes, which are estimated to be between 6- and 10-fold fewer in number compared to NP cells [36, 51], is unlikely to change overall conclusions about the effects of MMP-8 on nutrient diffusion.

A related consideration is the effects of MMP-8 treatment on cellular signaling. For example, MMP-8 expression has been shown to induce the expression of pro-inflammatory factors by breast cancer cells [52]. Also, the biologic effects of matrix fragments following proteolysis, which could activate toll-like receptors [53], may also be important. Having now established that matrix modification can enhance the transport properties of the CEP by enzymatic removal of matrix constituents *in vitro*, our current results motivate future studies to explore these biologic effects of MMP-8 treatment and matrix modification *in vivo*. In support, *in vivo* studies utilizing collagenases to increase tissue permeability showed minimal toxicity and off-target digestion at doses that increased solute uptake [54]. From a practical standpoint, targeted delivery of MMP-8 to the CEP could be achieved via injection under fluoroscopic guidance, and engineering approaches such as linking the enzyme to bulky nanoparticles could be used to control unwanted migration within the disc and to limit off-target activity.

Poor solute transport through the CEP impairs disc nutrition and could be a key factor that limits the success of intradiscal biologic therapies, which by design, increase nutrient demands. Thus, enhancing nutrient supply may be required to expand the application and utility of these emerging therapies, as well as inform alternative approaches for slowing or reversing degeneration. One strategy for enhancing nutrient supply involves targeted removal of proteins that impede solute transport in the CEP. Our current results showed that removal of CEP matrix constituents with MMP-8 increases solute uptake and nutrient diffusion *in vitro*. This effect was sensitive to AGE concentration, which limited solute uptake and susceptibility to MMP-8 activity. Taken together, these findings suggest that matrix reduction can enhance solute uptake and nutrient diffusion through the CEP, and that AGE concentration appears to be an important, patient-specific factor that influences the efficacy of this approach.

## Supporting information

S1 Fig Schematic of diffusion chamber for assessing the effects of MMP-8 on nutrient transport. The diffusion chambers consist of glass slides separated by impermeable spacers. Nucleus pulposus cells embedded in agarose gel obtain nutrients from their culture medium outside the chambers via diffusion through full-thickness human CEP sections. Following incubation, gels were stained to assess the viable distance from the open sides of the chamber.
(TIF)

S2 Fig MMP-8 Coomassie blue gel. MMP-8 fractions following size-exclusion chromatography.
(TIF)

S3 Fig MMP-8 treatment reduces CEP fixed charge density. CEP samples were treated for 18 h with MMP-8. Treatment-related loss of sGAG corresponded to decreases in calculated fixed charge density. Error bars represent ±SEM. One-way ANOVA with Tukey’s post-hoc test (*p* < 0.0001), ^a^*p* < 0.001, ^b^*p* < 0.01. *n* = 8 CEP samples per group, comprising two each from donors 1–4.
(TIF)

S4 Fig MMP-8 improves liposome uptake in CEP tissues. CEP samples treated for 18 h with 0.2 U/mL of MMP-8 show increased uptake of large liposomal nanoparticles. Each pair represents site-matched matched biopsy halves. *n* = 5 CEP samples per group, comprising one each from donors 2–4 and two from donor 1.
(TIF)

S5 Fig Collagen cross-linking restricts sodium fluorescein uptake in untreated CEP tissues. Untreated samples with high AGE concentration (>0.75 ng/μg collagen) exhibit restricted sodium fluorescein uptake. Each symbol represents a biopsy half from one of four donors. *t*-test, ^a^*p* = 0.0003.
(TIF)

S1 Table MMP-8 primer sets used for plasmid construction. (DOCX)

S2 Table CEP donor spine characteristics. (DOCX)
