# Supplementary figures and images for: Matrix modification for enhancing the transport properties of the human cartilage endplate to improve disc nutrition
Source: PLoS One. 2019 Apr 10;14(4):e0215218. doi: 10.1371/journal.pone.0215218 (PMC6457523; doi:10.1371/journal.pone.0215218)

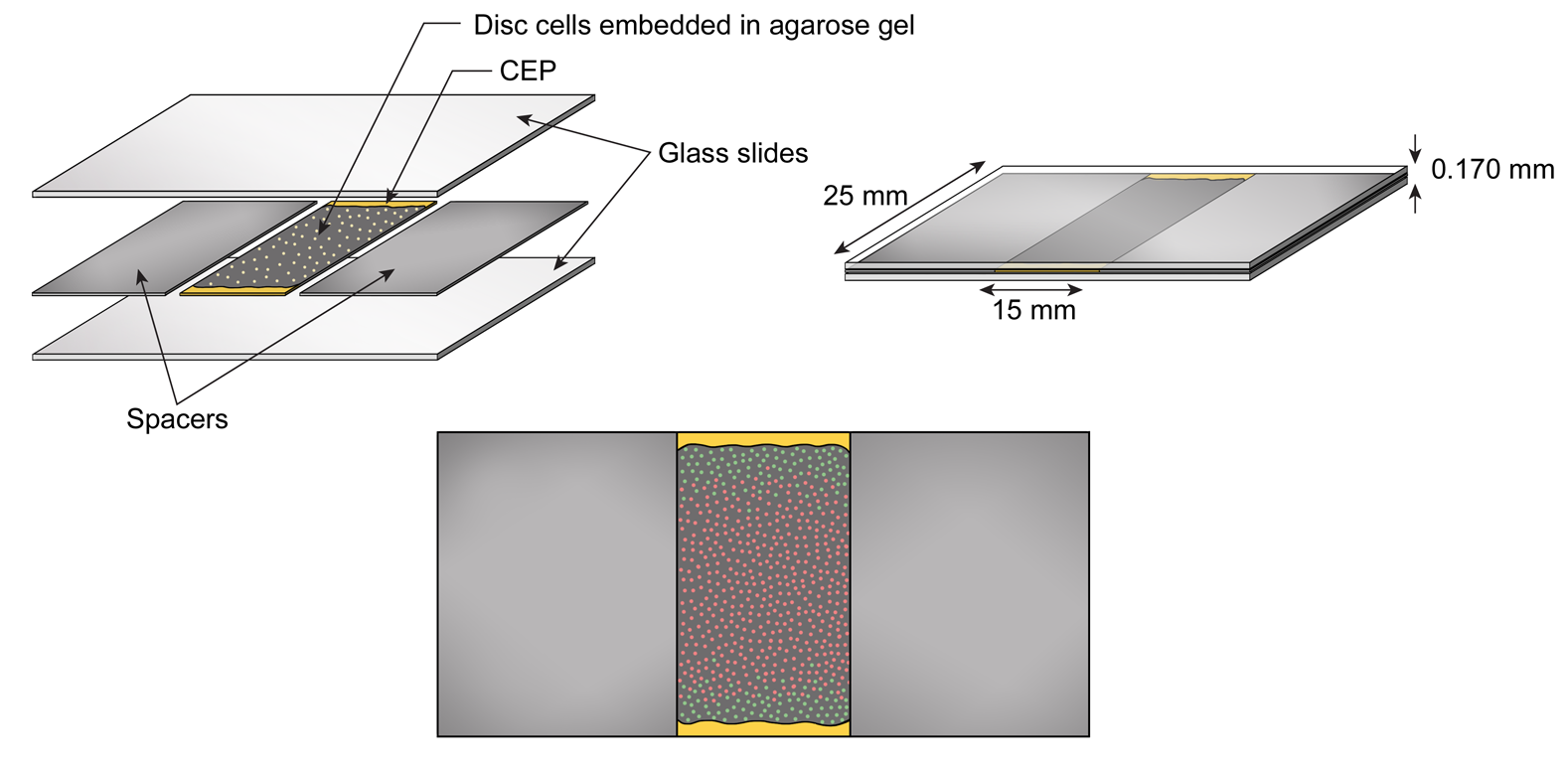

Supplement: S1 Fig — The diffusion chambers consist of glass slides separated by impermeable spacers. Nucleus pulposus cells embedded in agarose gel obtain nutrients from their culture medium outside the chambers via diffusion through full-thickness human CEP sections. Following incubation, gels were stained to assess the viable distance from the open sides of the chamber. (TIF) [file pone.0215218.s001.tif]

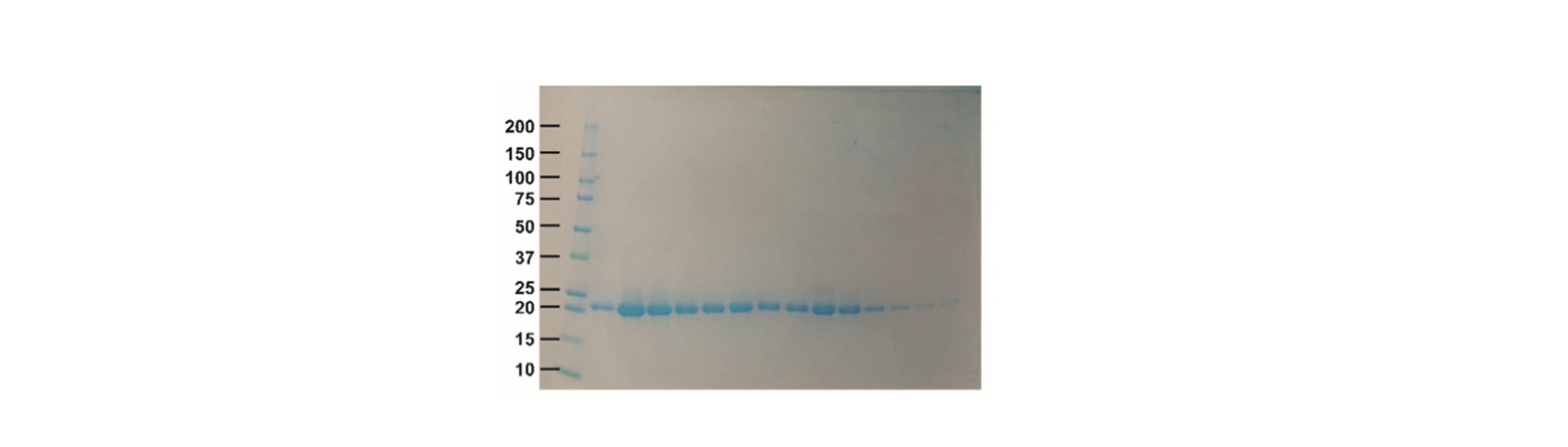

Supplement: S2 Fig — MMP-8 fractions following size-exclusion chromatography. (TIF) [file pone.0215218.s002.tif]

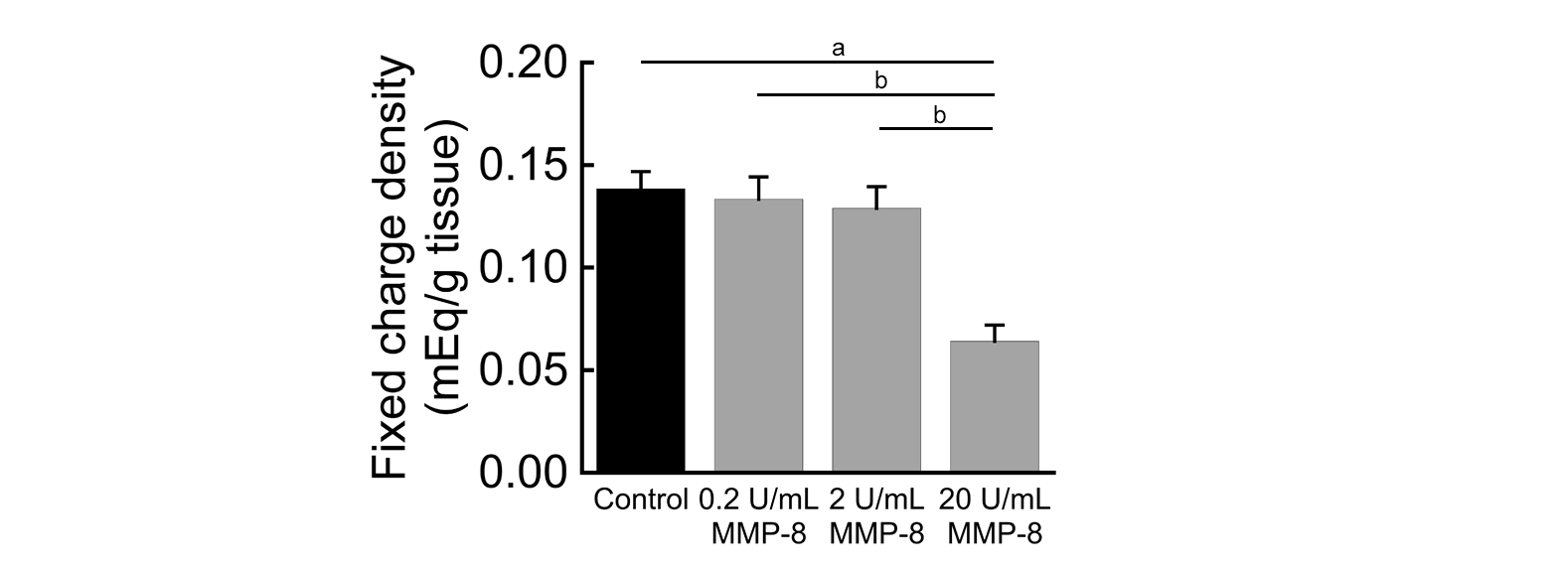

Supplement: S3 Fig — CEP samples were treated for 18 h with MMP-8. Treatment-related loss of sGAG corresponded to decreases in calculated fixed charge density. Error bars represent ±SEM. One-way ANOVA with Tukey’s post-hoc test (p < 0.0001), ap < 0.001, bp < 0.01. n = 8 CEP samples per group, comprising two each from donors 1–4. (TIF) [file pone.0215218.s003.tif]

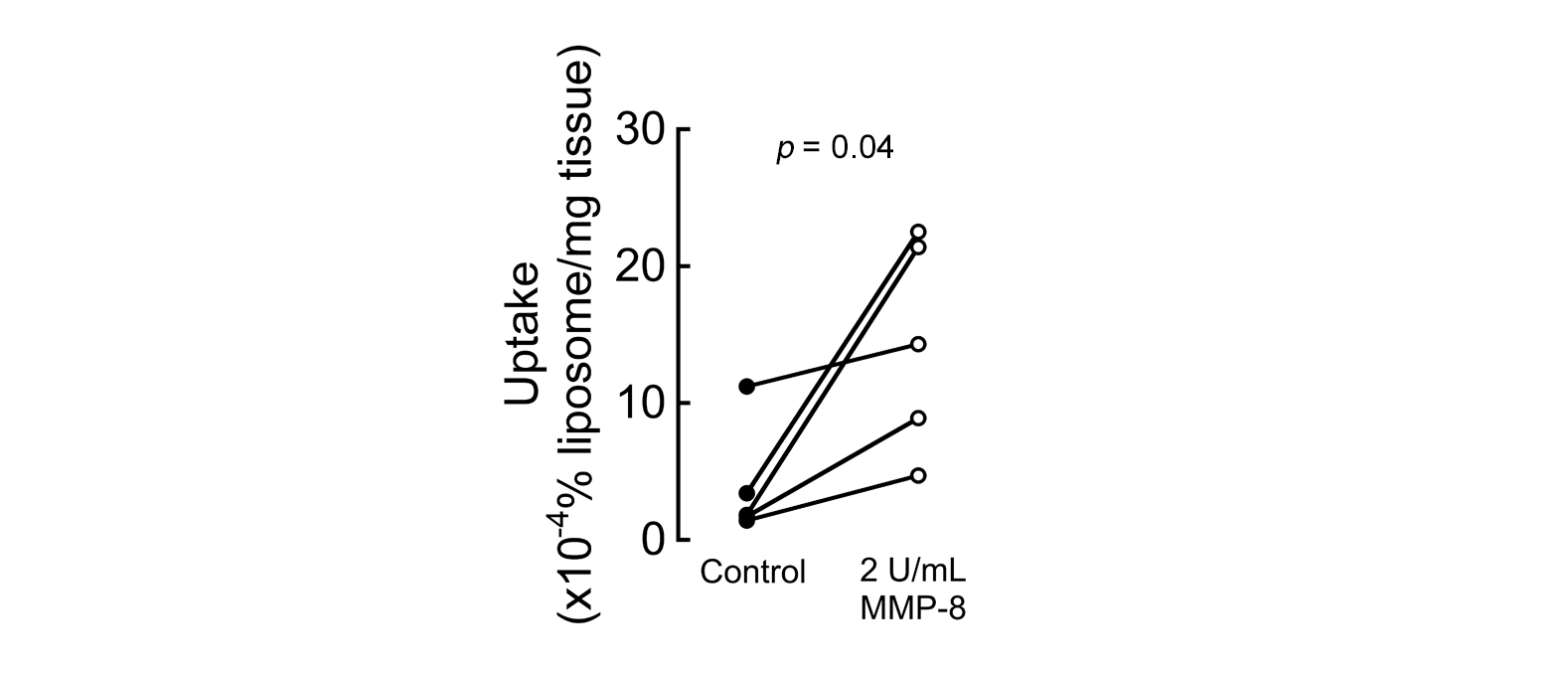

Supplement: S4 Fig — CEP samples treated for 18 h with 0.2 U/mL of MMP-8 show increased uptake of large liposomal nanoparticles. Each pair represents site-matched matched biopsy halves. n = 5 CEP samples per group, comprising one each from donors 2–4 and two from donor 1. (TIF) [file pone.0215218.s004.tif]

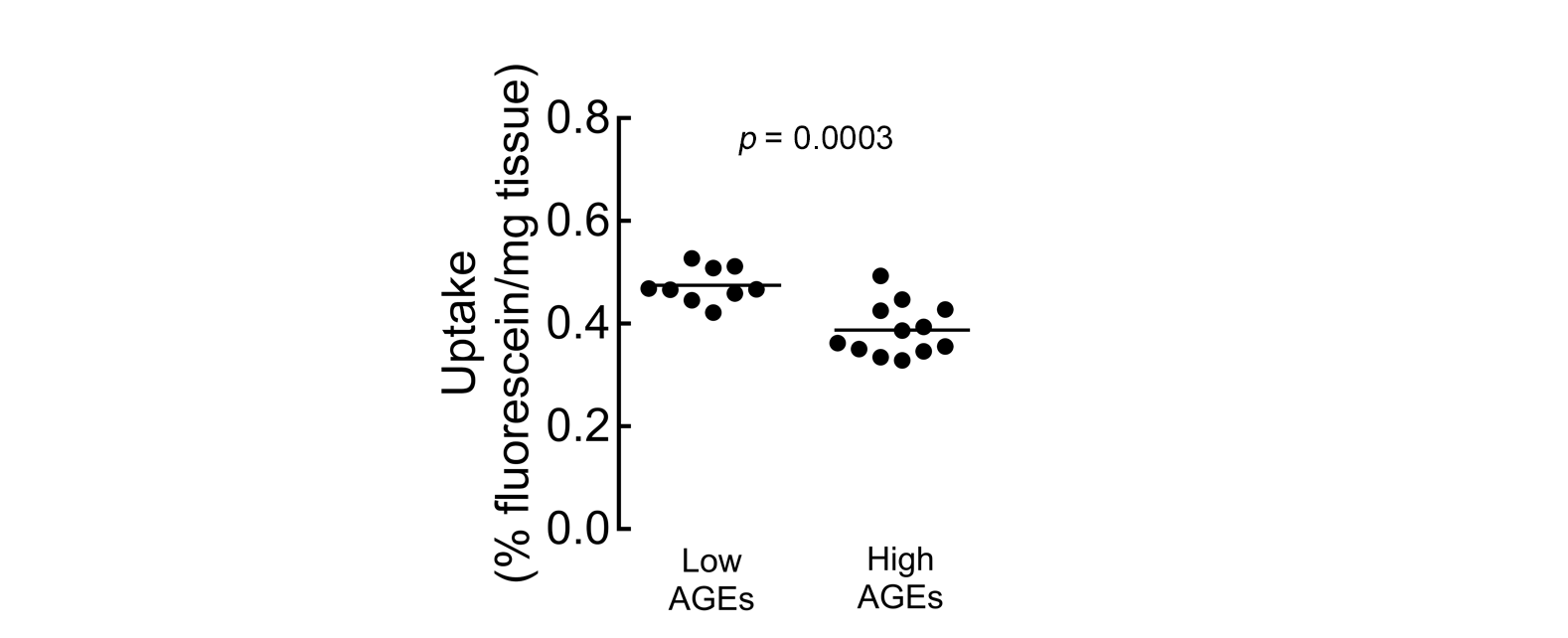

Supplement: S5 Fig — Untreated samples with high AGE concentration (>0.75 ng/μg collagen) exhibit restricted sodium fluorescein uptake. Each symbol represents a biopsy half from one of four donors. t-test, ap = 0.0003. (TIF) [file pone.0215218.s005.tif]
