# Supplementary material for: Matrix modification for enhancing the transport properties of the human cartilage endplate to improve disc nutrition
Source: PLoS One. 2019 Apr 10;14(4):e0215218. doi: 10.1371/journal.pone.0215218 (PMC6457523; doi:10.1371/journal.pone.0215218)
Supplement: S1 Table — (DOCX) [file pone.0215218.s006.docx]

**S1 Table.** **MMP-8 primer sets used for plasmid construction.**

| **Construct** | **#** | **Sequence** |
| --- | --- | --- |
| pET22B MMP-8 | 1 | TTGGGGTTTCCTGGGGTTAACATGGCCATCGCCGGCTGGGCAG (Vector-R) AGGCCATCTATGGACACCACCACCACCACCACTGAGATCC (Vector-F) CTCCTCGCTGCCCAGCCGGCGATGGCCATGTTAACCCCAGGAAACCCC (cDNA- F) ATCTCAGTGGTGGTGGTGGTGGTGTCCATAGATGGCCTGAATGCCATCGATGTC (cDNA-R) |
| pET22B MMP-8 GGS | 2 | GGCGGTTCTCACCACCACCACCACCACTGAGATCC - (F- 5’ Phosphorylated) TCCATAGATGGCCTGAATGCCATCGATGTC (R – 5’ Phosphorylated ) |
| pET22B MMP-8 PelB Removal | 3 | ATGTATATCTCCTTCTTAAAGTTAAACAAAATTATTTCTAGAGGGGAATTG (R-5’ Phosphorylated) ATGTTAACCCCAGGAAACCCCAAGTGGGAA (F-5’ Phosphorylated) |
