# Supplementary material for: Matrix modification for enhancing the transport properties of the human cartilage endplate to improve disc nutrition
Source: PLoS One. 2019 Apr 10;14(4):e0215218. doi: 10.1371/journal.pone.0215218 (PMC6457523; doi:10.1371/journal.pone.0215218)
Supplement: S2 Table — (DOCX) [file pone.0215218.s007.docx]

**S2 Table.** **CEP donor spine characteristics**

|  | **Age (yr)** | **Sex** | **Height (in)** | **Weight (lbs)** | **Pfirrmann grade (L4, L5)** | **Cause of death** |
| --- | --- | --- | --- | --- | --- | --- |
| Donor 1 | 66 | Female | 64 | 130 | III, III | Cirrhosis of liver, hypertension |
| Donor 2 | 52 | Male | 72 | 185 | IV, IV | COPD |
| Donor 3 | 38 | Male | 72 | 200 | II, II | Asphyxiation |
| Donor 4 | 60 | Female | 67 | 195 | III, III | Heart attack, chronic kidney disease |
